# COVID-19 social interventions were associated with amplified, not attenuated, indoor source pollutant–respiratory health associations: a six-year time-series analysis in Xi’an, China

**DOI:** 10.3389/fpubh.2026.1896806

**Published:** 2026-07-27

**Authors:** Shikang Liu, Zhaozhao Hui, Wen Zhang, Luping Chen, Mingxu Wang

**Affiliations:** 1 The First Affiliated Hospital of Xi’an Jiaotong University, Xi’an, China; 2 School of Public Health, Xi’an Jiaotong University, Xi’an, China

**Keywords:** air pollution, air quality health index, COVID-19, generalized additive model, natural experiment, respiratory diseases, time-series analysis, Xi’an

## Abstract

**Background:**

The COVID-19 pandemic triggered unprecedented social interventions that altered ambient air quality, population behavior, and healthcare-seeking. Whether these interventions modified the short-term association between air pollutants and respiratory health, and in which direction, remains unclear.

**Methods:**

We conducted a time-series study of daily respiratory outpatient visits (ICD-10: J00–J99, R04–R07, and R09) from a tertiary hospital in Xi’an, China, from January 2020 to December 2025. The study period was stratified into three COVID-19 phases: strict control (2020–2022), transition (Jan–Jun 2023), and post-pandemic (Jul 2023–2025). Generalized additive models with quasi-Poisson regression evaluated associations between PM2.5, SO2, NO2, and O3 and outpatient visits, adjusting for meteorological confounders, temporal trends, and phase. Pollutant-by-phase interaction terms tested for effect modification.

**Results:**

A total of 2,052,777 respiratory outpatient visits were recorded over 2,192 days. In full-period models, SO2 (lag4: ER = 9.11, 95% CI: 3.86–14.64%) and NO2 (lag5: ER = 2.72, 95% CI: 2.05–3.40%) showed the strongest associations. Phase-stratified analysis showed that the SO2 association was stronger during the strict control period (ER = 18.40, 95% CI: 10.29–27.11%) than the post-pandemic period (ER = 2.91, 95% CI: −4.82–11.28%), and the NO2 association followed the same pattern (3.07% vs. 1.31%). Formal interaction tests supported this gradient (SO2 × phase *p* = 0.13, NO2 × phase *p* = 0.15) but did not reach statistical significance, whereas PM2.5 and O3, which lack a dominant indoor combustion source, showed essentially no evidence of interaction (both *p* > 0.9). A localized Air Quality Health Index identified NO2 (55.4%) and SO2 (36.5%) as the dominant contributors to respiratory health risk.

**Conclusion:**

Contrary to the assumption that lockdowns would uniformly attenuate pollution–health effects, the health associations of combustion-source pollutants (SO2, NO2) in Xi’an were consistently stronger during strict COVID-19 control than the post-pandemic period, though formal interaction tests did not reach statistical significance. This pattern is consistent with, but does not confirm, an indoor exposure amplification mechanism in which prolonged home confinement increased cumulative exposure to residential heating emissions. These findings should be regarded as hypothesis-generating and indicate that reduced outdoor air pollution does not automatically translate into reduced health risk when population behavior changes substantially.

## 1 Introduction

Air pollution remains one of the most significant environmental risk factors for respiratory morbidity and mortality worldwide (1). The World Health Organization estimates that ambient air pollution contributes to approximately 4.2 million premature deaths annually (2), with respiratory diseases accounting for a substantial proportion of this burden (3). Epidemiological studies across diverse geographical settings have consistently demonstrated that short-term elevations in particulate matter (PM2.5), sulfur dioxide (SO2), nitrogen dioxide (NO2), and ozone (O3) are associated with increased healthcare utilization for respiratory conditions (4–7). Generalized additive models (GAM) with quasi-Poisson regression have become the methodological standard for quantifying these exposure–response relationships in time-series designs (8).

The COVID-19 pandemic, which began in late 2019 (9), created an unprecedented natural experiment for environmental health research. Governments imposed stringent non-pharmaceutical interventions, including lockdowns, factory closures, traffic restrictions, and mandatory mask-wearing (10), that dramatically reduced anthropogenic emissions (11). In China, the “dynamic zero-COVID” strategy, maintained until December 2022, involved repeated citywide lockdowns, mass testing, and strict quarantine that profoundly reshaped both ambient air quality and human exposure pathways (12). Most existing studies either focused on the pandemic’s direct respiratory effects (13) or examined pollution changes in isolation (14), without systematically evaluating how pandemic phases modified the pollution–health relationship (15). Whether lockdowns, by confining populations indoors with continued heating-source emissions, might paradoxically amplify the health effects of certain pollutants has not, to our knowledge, been directly examined.

Xi’an, the capital of Shaanxi Province in northwestern China, presents a particularly informative study setting. With a population exceeding 13 million, the city is situated in the Guanzhong Plain, a semi-enclosed basin where temperature inversions trap pollutants. Xi’an’s air pollution is characterized by the coexistence of coal combustion, vehicular emissions, and industrial discharges (16). The city experienced some of China’s most stringent lockdown measures, including a highly publicized citywide lockdown from December 23, 2021 to January 24, 2022. Importantly, residential coal and gas heating remains a major wintertime emission source, and prolonged home confinement during lockdowns may have increased indoor exposure to combustion-derived pollutants.

In this study, we leveraged the temporal structure of the COVID-19 pandemic to examine how social interventions modulated air pollution–respiratory health associations. Using 6 years (2020–2025) of daily outpatient data from a tertiary hospital in Xi’an, combined with ambient air quality and meteorological data, we pursued three objectives: (1) to quantify the lag effects of major pollutants on respiratory disease outpatient visits while controlling for pandemic phase; (2) to compare exposure–response relationships between the strict control period and the post-pandemic period through phase-stratified GAM models; and (3) to construct a localized Air Quality Health Index (AQHI) and evaluate pollutant-specific contributions to health risk.

## 2 Materials and methods

### 2.1 Study area

Xi’an is located in the central Guanzhong Plain in northwestern China (107°40′–109°49′E, 33°42′–34°45′N), covering approximately 10,108 km^2^ with a permanent population of 13.08 million. The city has a warm temperate semi-humid continental monsoon climate with four distinct seasons. The annual mean temperature ranges from 13.5 to 14.9 °C. The city’s basin topography, combined with intensive industrial activity, dense vehicular traffic, and wintertime residential heating, creates conditions conducive to the accumulation of atmospheric pollutants.

### 2.2 Data collection

*Respiratory disease data*. Daily outpatient visit records for respiratory diseases were obtained from the Hospital Information System (HIS) of a tertiary Grade A hospital in Xi’an, covering January 1, 2020 to December 31, 2025 (2,192 days). Records across all clinical departments (including respiratory medicine, otolaryngology, pediatrics, and emergency medicine) were included if the primary diagnosis corresponded to ICD-10 codes J00–J99 (diseases of the respiratory system), R04–R07 (respiratory symptoms), or R09.0–R09.3 (other respiratory symptoms). The daily count of outpatient visits served as the health outcome variable.

*Air pollution data*. Daily mean concentrations of six criteria pollutants—PM2.5, PM10, SO2, NO2, O3, and CO—were obtained from the China National Environmental Monitoring Centre, based on 24-h averages recorded at monitoring stations distributed across Xi’an.

*Meteorological data*. Daily meteorological variables, including mean temperature (°C), relative humidity (%), surface pressure (hPa), and wind speed (m/s), were obtained from the NASA Prediction of Worldwide Energy Resources (POWER) project for the Xi’an coordinates (34.26°N, 108.94°E).

### 2.3 COVID-19 pandemic phase classification

The study period was divided into three phases. The strict control period (January 2020–December 2022, *n* = 1,096 days) encompassed China’s “dynamic zero-COVID” strategy, during which Xi’an implemented multiple lockdowns, mass testing, workplace closures, and universal mask mandates. The transition period (January–June 2023, *n* = 181 days) represented the rapid policy shift following the “New Ten Measures” in December 2022. The post-pandemic period (July 2023–December 2025, *n* = 915 days) represented the return to pre-pandemic social and economic norms.

### 2.4 Statistical analysis

Given the non-normal distribution of daily outpatient visits and pollutant concentrations, descriptive statistics were reported as median (P25 and P75). Spearman rank correlation coefficients were used to assess pairwise associations among all variables.

Generalized additive models (GAM) based on quasi-Poisson regression were employed to analyze associations between pollutant concentrations and outpatient visits (8). The core model was: log[E(Yt)] = βXt + *γ*·COVID_phase + s(time, 6df/year) + s(temperature, 6df) + s(humidity, 3df) + DOW + holiday. Five strategies were employed: (1) full-period models with phase covariates; (2) phase-stratified models fitted separately for strict control and post-pandemic periods; (3) dual-pollutant models; (4) interaction models that included a pollutant × phase product term within a single regression, with the significance of the interaction coefficient assessed by a dispersion-adjusted Wald test, to formally evaluate whether the phase-stratified differences represented statistically significant effect modification rather than sampling variation between two independently fitted models; and (5) an exploratory comparison, restricted to the strict control period, of pollutant effects between the heating season (November–March) and the remaining months, to test whether the pattern of effect modification was consistent with a residential heating source. The excess risk (ER) per 10 μg/m^3^ increase was calculated as ER(%) = [exp(*β* × 10) − 1] × 100.

A localized Air Quality Health Index (AQHI) (17) was constructed using the cumulative excess risk from pollutants with significant positive associations: AQHI = 10/54.87 × *Σ*[exp(βi × Pi) − 1], where the summation is over PM2.5, SO2, and NO2 (18). Sensitivity analyses varied the degrees of freedom for the time-trend spline from 5 to 9 per year and excluded the transition period.

## 3 Results

### 3.1 Descriptive analysis

From 2020 to 2025, a total of 2,052,777 respiratory disease outpatient visits were recorded across 2,192 study days (Table 1). The daily median number of visits was 844 (P25: 411, P75: 1,416). The median daily concentrations of PM2.5, PM10, NO2, SO2, and O3 were 33.67, 75.94, 33.67, 6.50, and 52.81 μg/m^3^, respectively. Based on China’s GB 3095–2012 standard, the pollutant exceedance rates were PM2.5 (17.84%, 391 days), PM10 (13.50%, 296 days), NO2 (1.37%, 30 days), and SO2 and O3 (0%).

**Table 1:** Descriptive statistics of respiratory outpatient visits, air pollutants, and meteorological factors in Xi’an, 2020–2025.

Variable	Min	P25	Median	P75	Max	Mean ± SD
Visits (n/day)	8	411	844	1,416	2,777	936.49 ± 605.52
PM2.5 (μg/m^3^)	4.54	20.99	33.67	60.63	294.42	47.27 ± 39.64
PM10 (μg/m^3^)	8.46	47.28	75.94	119.13	1116.46	92.36 ± 72.71
NO2 (μg/m^3^)	6.88	21.99	33.67	48.09	93.75	36.37 ± 17.36
SO2 (μg/m^3^)	1.42	5.38	6.50	8.45	27.21	7.12 ± 2.97
O3 (μg/m^3^)	4.79	31.37	52.81	77.62	152.08	56.53 ± 30.58
Temperature (°C)	−8.44	5.91	15.15	23.89	35.17	14.86 ± 9.95
Humidity (%)	22.76	54.54	63.99	74.88	92.44	64.31 ± 13.37
Pressure (hPa)	930.80	940.10	947.75	954.30	972.00	947.55 ± 8.49
Wind speed (m/s)	0.40	1.03	1.37	1.91	5.34	1.54 ± 0.70

**Figure 1:**
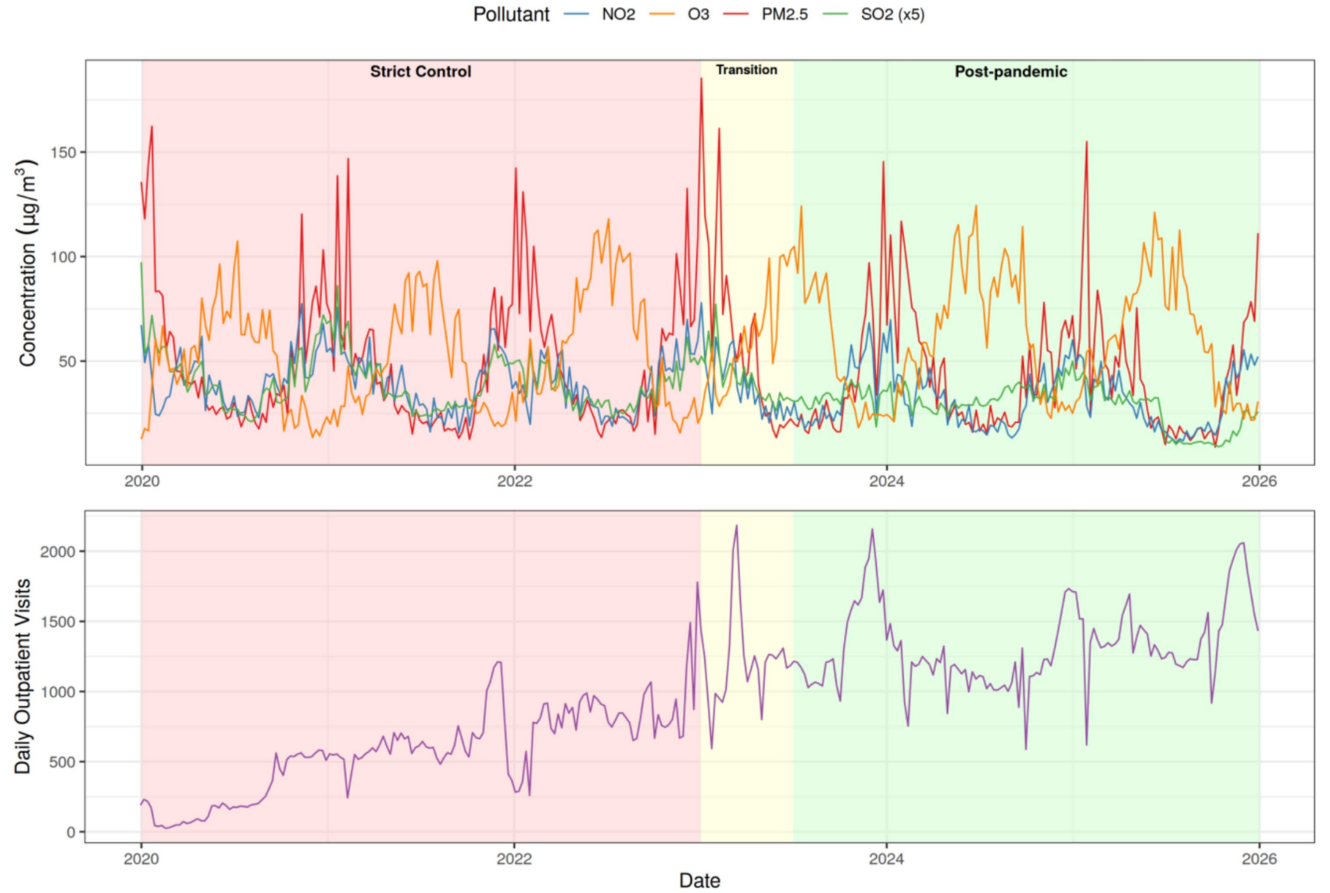
Time-series overview of weekly averaged air pollutant concentrations and respiratory outpatient visits in Xi’an, 2020–2025. Colored bands: strict control (pink), transition (yellow), post-pandemic (green).

Phase-stratified analysis showed that daily outpatient visits were substantially lower during the strict control period (median: 578, n = 1,096 days) than the post-pandemic period (median: 1,417, n = 915 days). PM2.5 and NO2 concentrations were paradoxically higher during strict control (PM2.5 median: 35.2 vs. 30.1; NO2 median: 38.2 vs. 27.8 μg/m^3^), consistent with continued heating emissions and winter inversion trapping (Figure 1).

### 3.2 Correlation analysis

Spearman correlation analysis revealed significant correlations among all pollutants (*p* < 0.05). PM10 and PM2.5 showed the strongest positive correlation, followed by PM10 and NO2, suggesting common emission sources. Given the high collinearity between PM10 and PM2.5, PM10 was excluded from subsequent modeling, retaining PM2.5, SO2, NO2, and O3 (Supplementary Figure S1).

### 3.3 Full-period single-pollutant model results

**Figure 2:**
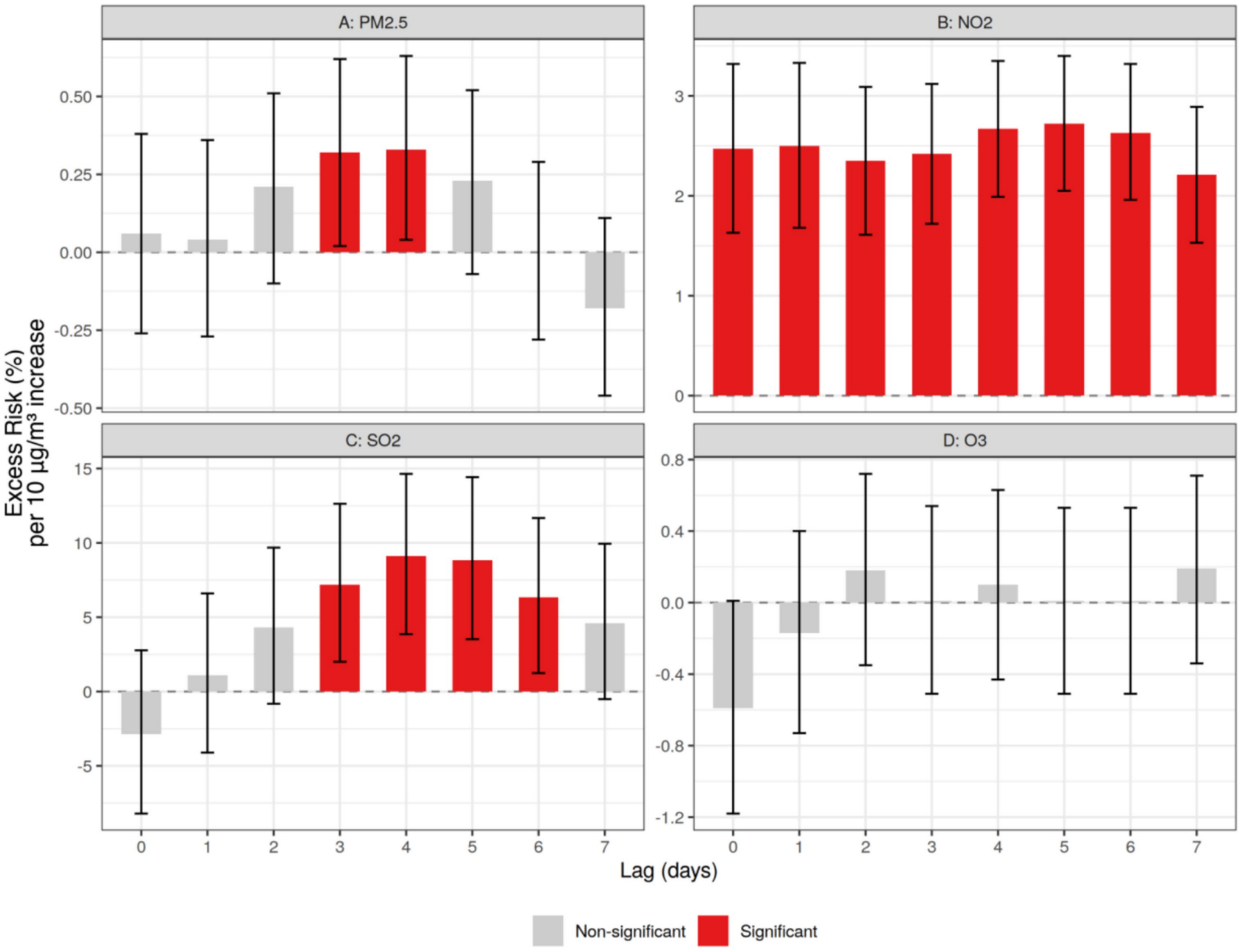
Excess risk (%) and 95% CIs of respiratory outpatient visits per 10 μg/m^3^ increase at lag0–lag7. A: PM2.5; B: NO2; C: SO2; D: O3. Red bars indicate *p* < 0.05.

After controlling for pandemic phase, temporal trends, meteorological conditions, and day-of-week effects (Figure 2), SO2 and NO2 demonstrated statistically significant positive associations with respiratory outpatient visits.

*SO2* exhibited the strongest per-unit effects, with significant single-day lag effects at lag3–lag6. The maximum single-day effect occurred at lag4: each 10 μg/m^3^ increase was associated with a 9.11% (95% CI: 3.86–14.64%) increase in outpatient visits. The cumulative effect at lag07 was 16.95% (95% CI: 6.98–27.85%).

*NO2* showed the most consistent effects, with statistically significant associations across all lag days (lag0–lag7). The maximum single-day effect was at lag5 (ER = 2.72, 95% CI: 2.05–3.40%) and the maximum cumulative effect at lag07 (ER = 8.22, 95% CI: 6.90–9.56%).

*PM2.5* showed significant effects only at lag3 (ER = 0.32%) and lag4 (ER = 0.33%). *O3* did not demonstrate statistically significant effects at any lag.

### 3.4 Dual-pollutant model results

Dual-pollutant models (Table 2) revealed that NO2 effects remained robust when paired with any pollutant (ER 2.69–2.98%, all *p* < 0.05). SO2 effects were robust paired with PM2.5 (ER = 8.04%, *p* = 0.005) and O3 (ER = 9.48%, *p* < 0.001), but attenuated when paired with NO2 (ER = 0.59%, NS), indicating confounding between these combustion-source pollutants.

**Table 2:** Dual-pollutant model results: ER (%) per 10 μg/m^3^ increase at best-effect lag day.

Model	Variable	ER (95% CI)	*P*	Sig
PM2.5 + NO2	PM2.5 (lag4)	−0.26 (−0.58, 0.08)	0.131	
	NO2 (lag5)	2.98 (2.23, 3.75)	<0.001	*
PM2.5 + SO2	PM2.5 (lag4)	0.15 (−0.18, 0.47)	0.370	
	SO2 (lag4)	8.04 (2.37, 14.02)	0.005	*
PM2.5 + O3	PM2.5 (lag4)	0.34 (0.05, 0.64)	0.023	*
	O3 (lag0)	−0.63 (−1.22, −0.03)	0.039	*
NO2 + SO2	NO2 (lag5)	2.69 (1.95, 3.43)	<0.001	*
	SO2 (lag4)	0.59 (−4.69, 6.16)	0.831	
NO2 + O3	NO2 (lag5)	2.72 (2.05, 3.40)	<0.001	*
	O3 (lag0)	−0.62 (−1.20, −0.03)	0.039	*
SO2 + O3	SO2 (lag4)	9.48 (4.20, 15.03)	<0.001	*
	O3 (lag0)	−0.67 (−1.26, −0.07)	0.028	*

### 3.5 Phase-stratified analysis

**Figure 3:**
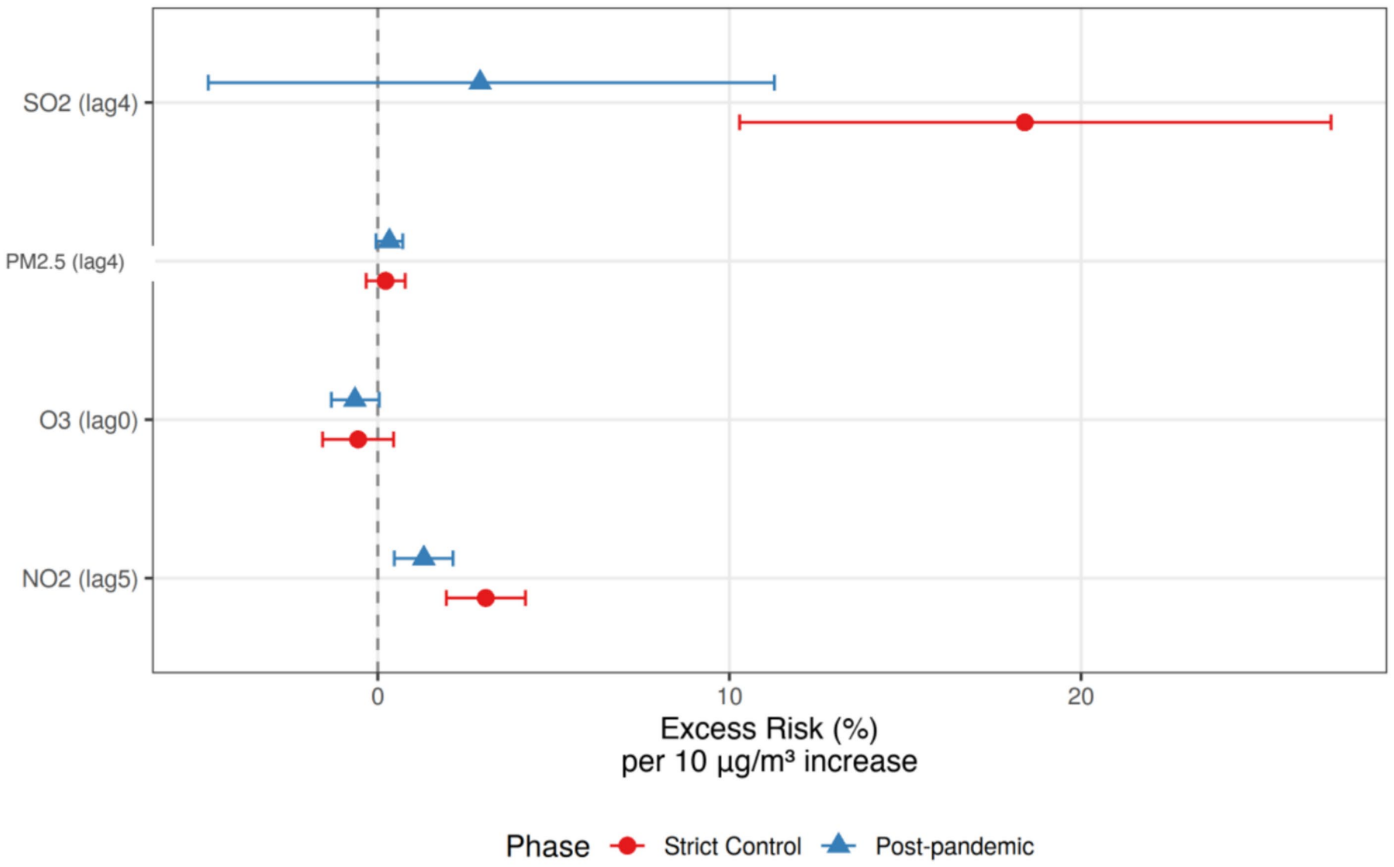
Forest plot comparing phase-stratified excess risk at each pollutant’s best-effect lag day. Red: strict control; Blue: post-pandemic. Error bars = 95% CI.

Phase-stratified GAM models revealed a striking pattern (Table 3, Figure 3). Contrary to the initial hypothesis that social interventions would attenuate pollution–health effects, the point estimates for combustion-source pollutants were consistently higher during the strict control period than during the post-pandemic period.

**Table 3:** Phase-stratified excess risk (%) per 10 μg/m^3^ increase: strict control vs. post-pandemic.

Pollutant	Lag	Strict Control ER (95% CI)	Post-pandemic ER (95% CI)	Ratio	Direction	Interaction P
SO2	lag4	18.40 (10.29, 27.11)*	2.91 (−4.82, 11.28)	6.3×	Higher in strict control	0.130
NO2	lag5	3.07 (1.95, 4.20)*	1.31 (0.47, 2.14)*	2.3×	Higher in strict control	0.153
PM2.5	lag4	0.22 (−0.33, 0.78)	0.33 (−0.05, 0.71)		NS both	0.979
O3	lag0	−0.56 (−1.57, 0.45)	−0.65 (−1.32, 0.04)		NS both	0.931

*P < 0.05. NS = not statistically significant. Interaction P = Wald test p-value for the pollutant × phase interaction term from a single quasi-Poisson generalized additive model fitted jointly to both phases, testing whether the strict control and post-pandemic estimates differ significantly from each other.

The SO2 effect at lag4 was more than six times higher during strict control (ER = 18.40, 95% CI: 10.29–27.11%) than during the post-pandemic period (ER = 2.91, 95% CI: −4.82–11.28%). Similarly, NO2 at lag5 was 2.3 times higher during strict control (ER = 3.07, 95% CI: 1.95–4.20%) than post-pandemic (ER = 1.31, 95% CI: 0.47–2.14%). PM2.5 and O3 were non-significant in both phases.

To determine whether these phase-stratified differences reflected statistically significant effect modification rather than sampling variation between two separately fitted models, we additionally fitted pollutant × phase interaction models within a single regression framework (Table 3). The interaction term approached, but did not reach, conventional statistical significance for SO2 (*p* = 0.13) and NO2 (*p* = 0.15). PM2.5 and O3, neither of which showed a phase difference in the stratified models, also showed no evidence of interaction (*p* = 0.98 and *p* = 0.93, respectively). A markedly lower interaction *p*-value for the two combustion-source pollutants than for the two pollutants without a dominant indoor source is consistent with effect modification concentrated among combustion-related pollutants, but the individual tests are not definitive on their own and the possibility of a chance finding cannot be excluded.

As an exploratory test of the proposed indoor-heating mechanism, we compared pollutant effects within the strict control period between the heating season (November–March, when centralized and household coal or gas heating operates in Xi’an) and the remaining months. The SO2 effect was higher during the heating season (ER = 22.30%) than outside it (ER = 15.65%), and the NO2 effect followed the same direction (4.30% vs. 2.60%), consistent with a residential heating source. Neither within-period comparison reached statistical significance (interaction *p* = 0.48 for SO2, *p* = 0.16 for NO2), and this analysis should be regarded as descriptive rather than confirmatory given the smaller sample size within each subgroup.

### 3.6 Sensitivity analysis

When the degrees of freedom for the time-trend spline were varied from 5 to 9 per year, SO2 and NO2 effects remained consistently significant. O3 remained non-significant across all specifications (Supplementary Figure S2). The phase-stratified comparison remained robust after excluding the transition period.

### 3.7 Air quality health index

**Figure 4:**
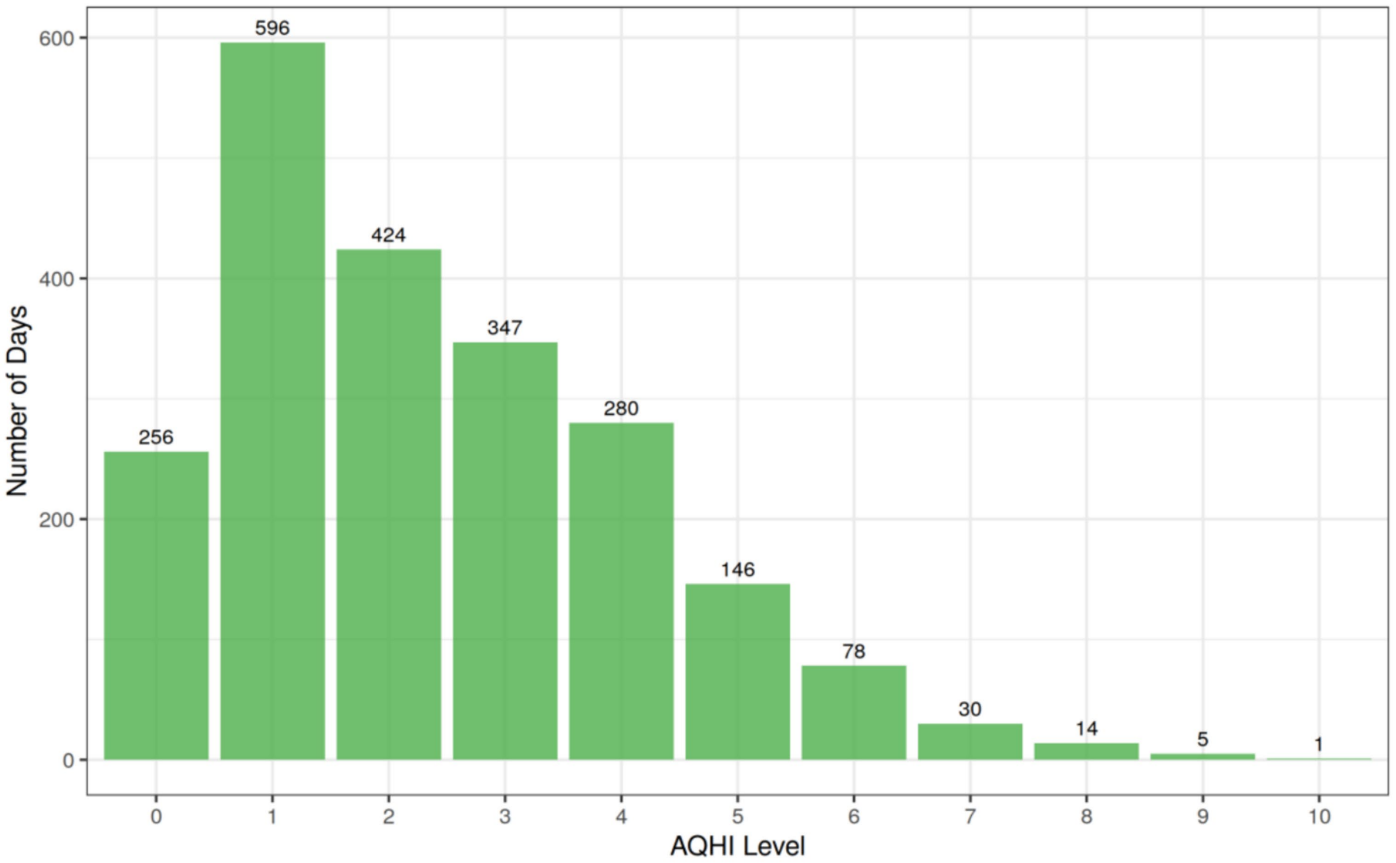
Distribution of the localized Air Quality Health Index (AQHI) in Xi’an, 2020–2025.

The localized AQHI was constructed using PM2.5, SO2, and NO2 (O3 excluded due to non-significant association). The formula was: AQHI = 10/54.87 × [exp(0.000333 × PM2.5) + exp(0.008722 × SO2) + exp.(0.002684 × NO2) − 3]. The distribution was right-skewed, with 93.9% of days having AQHI < 6 (Figure 4). NO2 contributed the most to excess risk (55.4%), followed by SO2 (36.5%) and PM2.5 (8.1%).

## 4 Discussion

This study is, to our knowledge, among the first to examine whether COVID-19 social interventions were associated with amplified rather than attenuated health effects of combustion-source air pollutants on respiratory outpatient visits. Using 6 years of daily data from Xi’an spanning the complete pandemic trajectory, we found that the point estimates for SO2 and NO2 were 2 to 6 times higher during the strict control period than the post-pandemic period, although the corresponding pollutant × phase interaction tests did not reach statistical significance.

### 4.1 The indoor exposure amplification hypothesis

One possible explanation for the stronger SO2–health association observed during strict control is an indoor exposure amplification effect. During Xi’an’s prolonged lockdowns, residents spent most of their time indoors. In northern Chinese cities, wintertime residential heating, predominantly from centralized coal-fired boilers and individual coal stoves, is a major source of SO2. Lockdowns reduced vehicular and industrial emissions (19) but did not reduce heating-related emissions, so the combination of sustained indoor SO2 exposure, reduced ventilation, and longer time spent at home could plausibly have raised cumulative personal exposure even where ambient outdoor concentrations were lower. We emphasize that this remains a hypothesis: our dataset does not include indoor air quality measurements, ventilation practices, heating fuel use, or time-activity data, and the interaction tests reported above did not reach statistical significance. The exploratory heating-season comparison, in which the SO2 and NO2 effects trended higher during the heating months than during the rest of the strict control period, offers a pattern consistent with this mechanism, but it is not a direct test of indoor exposure and cannot rule out other explanations.

The pattern across pollutants is broadly consistent with this interpretation, though we present it as circumstantial rather than confirmatory. SO2, which is strongly tied to coal combustion, showed both the largest point estimate for amplification and the lowest interaction *p*-value among the four pollutants. NO2, which originates from both traffic and heating sources, showed an intermediate pattern. PM2.5, which has more heterogeneous sources (20) including secondary formation (21), showed no phase difference, and O3, which forms outdoors through photochemical reactions and cannot accumulate indoors (22), showed no association in either phase and an interaction *p*-value close to 1. A gradient running from the strongest signal for the indoor combustion product to essentially no signal for the purely outdoor photochemical pollutant is the pattern one would expect if indoor exposure amplification were occurring, but it is also a pattern that alternative explanations, including differential confounding by season or by healthcare-seeking behavior across pollutants, could in principle produce. We therefore regard the indoor exposure amplification hypothesis as a plausible and mechanistically coherent explanation that is supported by, but not established by, the present data.

### 4.2 NO2: traffic reduction was insufficient

The stronger NO2 association during strict control, despite substantial traffic reduction, merits separate consideration. Vehicular emissions are the primary source of ambient NO2, but residential gas cooking and heating also generate indoor NO2, and the frequency of indoor cooking plausibly increased once restaurants closed during lockdowns. Ambient NO2 concentrations were in fact higher during strict control (median: 38.2 μg/m^3^) than post-pandemic (median: 27.8 μg/m^3^), suggesting that heating emissions and winter temperature inversions offset the reduction in traffic-related emissions at the ambient level. This observation helps explain why NO2 concentrations did not fall as much as expected, but it does not by itself establish that indoor exposure, rather than the higher ambient concentration alone, drove the stronger health association.

### 4.3 Comparison with existing literature

Our findings diverge from studies reporting attenuated pollution–health associations during lockdowns (23). This discrepancy likely reflects differences in study setting, pollutant mix, and lockdown stringency (24). Most prior studies were conducted in regions where residential heating is not coal-dependent or where lockdowns were shorter (22). In Xi’an, the combination of prolonged lockdowns (nearly 3 years), harsh winters requiring intensive heating, and basin topography that traps emissions created a unique environment where indoor exposure amplification dominated.

### 4.4 Policy implications

If confirmed by future studies incorporating direct exposure measurements, these findings would have three policy implications. First, future pandemic preparedness plans should incorporate indoor air quality monitoring and ventilation guidelines into lockdown protocols, particularly in regions reliant on combustion-based heating. Second, the AQHI analysis identified NO2 as the dominant contributor to respiratory health risk (55.4%), suggesting that emission reduction strategies should prioritize traffic management and clean heating transitions regardless of the amplification hypothesis. Third, the pronounced phase-dependent variation suggests that static AQHI weights may underestimate health risks during periods of substantial behavioral change (25), an issue that merits further investigation.

### 4.5 Limitations

Several limitations should be acknowledged. First, outpatient data were from a single tertiary hospital, which limits generalizability and means that shifts in referral patterns or care-seeking behavior at that hospital could influence the results independently of pollutant exposure. Second, ambient monitoring station concentrations are an imperfect proxy for personal exposure. We had no direct measurements of indoor air quality, ventilation practices, heating fuel type, or time-activity patterns, and the indoor exposure amplification hypothesis therefore cannot be directly tested with the present data; it should be treated as a plausible interpretation of an observed pattern rather than a demonstrated mechanism. Third, healthcare-seeking behavior changed substantially during the pandemic (13). Although our phase-stratified and interaction models compare within-phase associations rather than raw visit counts, we cannot fully exclude the possibility that changes in who sought care, and for what severity of symptoms, differed systematically between phases in ways that mimic effect modification. Fourth, the formal pollutant × phase interaction tests for SO2 and NO2 did not reach statistical significance, and the exploratory heating-season comparison was likewise non-significant; both analyses are directionally consistent with the amplification pattern but do not provide confirmatory evidence, and the possibility of a chance finding cannot be excluded given the number of comparisons performed. Fifth, as a single-city study conducted in a coal-heating-dependent region, generalizability to cities with different heating infrastructure, climate, or lockdown stringency requires further investigation. Given these constraints, we present the indoor exposure amplification hypothesis as hypothesis-generating, and we recommend that future work incorporate personal exposure monitoring, indoor air quality measurement, and time-activity data to test it directly.

## 5 Conclusion

Leveraging the COVID-19 pandemic as a natural experiment, this study found that the short-term associations between combustion-source air pollutants (SO2, NO2) and respiratory disease outpatient visits in Xi’an, China, were consistently stronger during strict COVID-19 control than during the post-pandemic period. The SO2–health association was 6.3 times higher during strict control than post-pandemic, and the NO2 association was 2.3 times higher, although formal interaction tests for both pollutants stopped short of conventional statistical significance. These findings are consistent with, but do not confirm, an indoor exposure amplification hypothesis, and we present them as hypothesis-generating. Direct exposure studies are needed before this pattern can inform pandemic preparedness or air pollution governance strategies with confidence.

## Data Availability

The original contributions presented in the study are included in the article/Supplementary material, further inquiries can be directed to the corresponding author.
